# Redetermination of di-μ-hydroxido-bis­[diaqua­chlorido­dioxido­uranium(VI)] from single-crystal synchrotron data

**DOI:** 10.1107/S1600536810002394

**Published:** 2010-01-23

**Authors:** Diederik Huys, Rik Van Deun, Phil Pattison, Luc Van Meervelt, Kristof Van Hecke

**Affiliations:** a Department of Chemistry, Katholieke Universiteit Leuven, Celestijnenlaan 200F, B-3001 Leuven (Heverlee), Belgium; b Inorganic and Physical Chemistry Group, Ghent University, Krijgslaan 281 - Building S3, B-9000 Gent, Belgium; c Swiss–Norwegian Beamline (SNBL), European Synchrotron Radiation Facility (ESRF), Rue Jules Horowitz, F-38043 Grenoble, France

## Abstract

The title compound, [(UO_2_)_2_Cl_2_(OH)_2_(H_2_O)_4_], was obtained unintentionally as the product of an attempted reaction between uranium(VI) oxide dihydrate, UO_3_·2H_2_O, and hydrogen bis­(trifluoro­methyl­sulfon­yl)imide (HTf_2_N), in an experiment to obtain crystals of uranyl bis­(trifluoro­methyl­sulfon­yl)imide, UO_2_(Tf_2_N)_2_·*x*H_2_O. The structure consists of neutral dimers of uranyl (UO_2_^2+^) units, double bridged by OH^−^ anions. Each uranyl unit is surrounded by one Cl and four O atoms, which form an irregular penta­gon, in a plane perpendicular to the linear uranyl groups. The coordination geometry around each U atom can be considered to be distorted penta­gonal-bipyramidal. In the crystal structure the uranyl dimers are connected to each other by hydrogen-bonding inter­actions [O⋯Cl = 3.23 (1) Å].

## Related literature

For general background to the use of uranyl bis­(trifluoro­methyl­sulfon­yl)imide as a starting material for the study of the spectroscopic properties of uranyl complexes in ionic liquids, see: Nockemann *et al.* (2007 ▶); Binnemans (2007 ▶). For the original published structure determined from Weissenberg data, see: Åberg (1969 ▶). For related structures, see: Åberg (1970 ▶); Tsushima *et al.* (2007 ▶). For databases of inorganic structures, see: Bergerhoff *et al.* (1983 ▶); ICSD (2009 ▶).
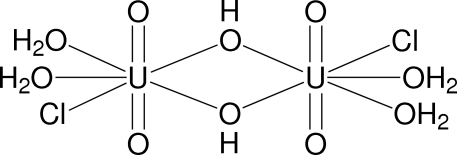


## Experimental

### 

#### Crystal data

[U_2_Cl_2_O_4_(OH)_2_(H_2_O)_4_]
*M* *_r_* = 717.04
Monoclinic, 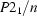

*a* = 10.712 (2) Å
*b* = 6.1212 (12) Å
*c* = 17.662 (4) Å
β = 95.47 (3)°
*V* = 1152.8 (4) Å^3^
*Z* = 4
Synchrotron radiation
λ = 0.77000 Å
μ = 63.63 mm^−1^
*T* = 100 K
0.15 × 0.10 × 0.1 mm

#### Data collection

ESRF, SNBL, BM01A diffractometer
Absorption correction: multi-scan (SCALE3 in *ABSPACK*; Oxford Diffraction, 2006 ▶) *T*_min_ = 0.008, *T*_max_ = 0.056
13020 measured reflections
1846 independent reflections
1620 reflections with *I* > 2σ(*I*)
*R*_int_ = 0.067

#### Refinement

*R*[*F* ^2^ > 2σ(*F* ^2^)] = 0.045
*wR*(*F* ^2^) = 0.131
*S* = 1.11
1846 reflections
127 parameters
H atoms not located
Δρ_max_ = 3.37 e Å^−3^
Δρ_min_ = −1.67 e Å^−3^

### 

Data collection: *MAR345 Program Manual* (Mar, 2000 ▶); cell refinement: *CrysAlis PRO* (Oxford Diffraction, 2006 ▶); data reduction: *CrysAlis PRO*; program(s) used to solve structure: *SHELXS97* (Sheldrick, 2008 ▶); program(s) used to refine structure: *SHELXL97* (Sheldrick, 2008 ▶); molecular graphics: *PLUTON* (Spek, 2009 ▶); software used to prepare material for publication: *PLATON* (Spek, 2009 ▶).

## Supplementary Material

Crystal structure: contains datablocks I, global. DOI: 10.1107/S1600536810002394/bt5153sup1.cif

Structure factors: contains datablocks I. DOI: 10.1107/S1600536810002394/bt5153Isup2.hkl

Additional supplementary materials:  crystallographic information; 3D view; checkCIF report

## Figures and Tables

**Fig. 1.**
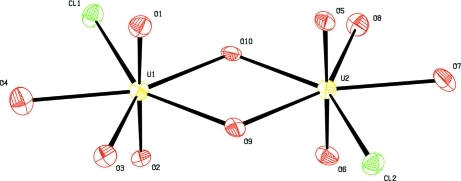
Coordination geometry of the title compound, showing the atom-labelling scheme of the asymmetric unit and 50% probability displacement ellipsoids.

**Fig. 2.**
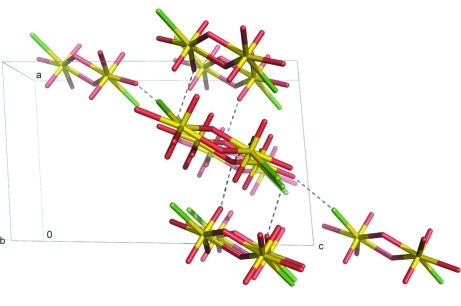
Packing diagram of the title compound, showing the hydrogen bonding between symmetry equivalent molecules.

## supplementary crystallographic information

### Comment

Uranyl bis(trifluoromethylsulfonyl)imide is a useful starting material for the study of the spectroscopic properties of uranyl complexes in ionic liquids (Nockemann *et al.*, 2007; Binnemans, 2007). The presence of chloride ions in the final product was surprising, because no chloride had been added to the reaction mixture. The chloride contamination of the reaction mixture can probably be attributed to chloride impurities in the aqueous hydrogen bis(trifluoromethylsulfonyl)imide solution. It should be noted that the presence of chloride traces in the HTf_2_N solution is not surprising, since the bis(trifluoromethylsulfonyl)imide anion can be synthesized by reaction between trifluorometylsulfonylamide and trifluoromethylsulfonyl chloride. Moreover, the uranyl ion has a strong tendency to form chloro complexes when both chloride ions and bis(trifluoromethylsulfonyl)imide ions are present (Nockemann *et al.*, 2007). The acidity of coordinated water molecules, especially those in bridging positions, easily leads to the formation of hydroxo dimers or oligomers in neutral aqueous medium (Åberg, 1970; Tsushima *et al.*, 2007).

The structure of the title compound [(UO_2_)_2_(OH)_2_Cl_2_(H_2_O)_4_] (Figure 1) is analogous to the structure as previously determined by the Weissenberg photographical technique (Åberg, 1969), with ICSD entry 31006 (ICSD Version 1.4.6) (Bergerhoff *et al.*, 1983; ICSD, 2009).

However, in the latter structure, no meaningful anisotropic refinement could be carried out for the positions of the oxygen atoms (Åberg, 1969).

The asymmetric unit consists of uranyl dimers, double bridged by OH^-^-anions. Each uranyl cation is surrounded by one chlorine and four oxygen atoms, which form an irregular pentagon, in a plane perpendicular to the linear uranyls. The coordination geometry around each uranium atom can be considered as a distorted pentagonal bipyramid (Figure 2). As in the structure of Åberg, no hydrogen atoms could be unambiguously located on the water molecules, nor on the hydroxyl anions.

The U1—U2 distance in a uranyl dimer is 3.949 (1) Å. U—Cl distances are 2.751 (3) Å and 2.772 (4) Å, for U1—Cl1 and U2—Cl2, respectively.

The uranyl U=O distances vary between 1.746 (9) Å and 1.790 (9) Å, while the two uranyl groups themselves are quasi-linear, with O=U=O angles of 177.7 (4)° and 177.8 (4)°, respectively.

The U—O distances vary between 2.37 (1) Å and 2.49 (1) Å. The U—O distances within the bridge formed by O9 and O10 range from 2.37 (1) to 2.382 (9) Å. The most lateral positioned oxygen atoms O4 and O7 show both a larger U—O distance of 2.49 (1) Å.

Notably, the *a*- and *c*-axis of the structure of Åberg are interchanged, compared to the reported structure, hence caution should be paid when comparing the crystal packing environments.

As in the previously reported structure (Åberg, 1969), hydrogen bonding is observed between the dimers, mainly extending in the (001)-plane in the [100] direction (Figure 3). These hydrogen bonds are directed from uranium-coordinating oxygen atoms to chloride atoms on a neighboring dimer: Cl1···O8 (3.08 (1) Å), Cl2···O3 (3.11 (1) Å) and between bridging hydroxyl oxygen atoms and uranyl oxygen atoms: O10···O2 (2.80 (1) Å), O9···O5 (2.87 (1) Å). The same bridging hydroxyl oxygen atoms O9 and O10 are further connected through hydrogen bonds in the [010] direction (*b*-direction) to the uranium coordinated oxygen atoms O8 (2.66 (1) Å) and O3 (2.65 (1) Å), respectively.

Only one hydrogen bond is observed between one of the lateral coordinating oxygen atoms O4 and the Cl2-atom of a subsequent dimer (3.23 (1) Å), which additionally links the dimers together in the [001] direction (*c*-direction).

### Experimental

Uranium(VI)oxide dihydrate UO_3_.2H_2_O (966 mg, 3 mmol), suspended in 10 ml of deionized water. To this suspension, 2 equivalents (2.4 g) of a 80% solution of hydrogen bis(trifluoromethylsulfonyl)imide (from IoLiTec) was added and the mixture was refluxed while stirred for 1 h. After leaving the solution to cool to room temperature, the excess of UO_3_ was filtered off and the clear liquid was evaporated to dryness using a rotary evaporator. The remaining thick slurry was further dried at 60 °C using a Schlenk apparatus for 36 h, yielding 885 mg of a glassy residue. A small solid sample was removed from the batch and the remaining solid was redissolved in a minimal amount of water, while slightly heating using a heat gun. The liquid was transferred to a small crystallization dish and cooled to 5 °C in a refrigerator. A very small amount of the dry solid product was then carefully added to the solution and the crystallization dish was put in a desiccator at room temperature. After 10 weeks, small hygroscopic crystals were obtained.

### Refinement

No hydrogen atoms could be unambiguously located on the water molecules, nor on the hydroxyl anions.

### Crystal data

**Table:**

[U_2_Cl_2_O_4_(OH)_2_(H_2_O)_4_]	*F*(000) = 1232
*M**_r_* = 717.04	*D*_x_ = 4.131 Mg m^−^^3^
Monoclinic, *P*2_1_/*n*	Synchrotron radiation, λ = 0.77000 Å
Hall symbol: -P 2yn	Cell parameters from 4751 reflections
*a* = 10.712 (2) Å	θ = 2.3–26.2°
*b* = 6.1212 (12) Å	µ = 63.63 mm^−^^1^
*c* = 17.662 (4) Å	*T* = 100 K
β = 95.47 (3)°	Block, yellow
*V* = 1152.8 (4) Å^3^	0.15 × 0.1 × 0.1 mm
*Z* = 4	

### Data collection

**Table:**

ESRF, SNBL, BM01A diffractometer	1846 independent reflections
Radiation source: bending magnet	1620 reflections with *I* > 2σ(*I*)
double crystal	*R*_int_ = 0.067
φ scans	θ_max_ = 26.4°, θ_min_ = 2.3°
Absorption correction: multi-scan (SCALE3 in ABSPACK; Oxford Diffraction, 2006)	*h* = −12→12
*T*_min_ = 0.008, *T*_max_ = 0.056	*k* = 0→7
13020 measured reflections	*l* = 0→20

### Refinement

**Table:**

Refinement on *F*^2^	0 restraints
Least-squares matrix: full	Primary atom site location: structure-invariant direct methods
*R*[*F*^2^ > 2σ(*F*^2^)] = 0.045	Secondary atom site location: difference Fourier map
*wR*(*F*^2^) = 0.131	*w* = 1/[σ^2^(*F*_o_^2^) + (0.0885*P*)^2^] where *P* = (*F*_o_^2^ + 2*F*_c_^2^)/3
*S* = 1.11	(Δ/σ)_max_ < 0.001
1846 reflections	Δρ_max_ = 3.37 e Å^−^^3^
127 parameters	Δρ_min_ = −1.67 e Å^−^^3^

### Special details

**Table:**

Geometry. All e.s.d.'s (except the e.s.d. in the dihedral angle between two l.s. planes) are estimated using the full covariance matrix. The cell e.s.d.'s are taken into account individually in the estimation of e.s.d.'s in distances, angles and torsion angles; correlations between e.s.d.'s in cell parameters are only used when they are defined by crystal symmetry. An approximate (isotropic) treatment of cell e.s.d.'s is used for estimating e.s.d.'s involving l.s. planes.
Refinement. Refinement of *F*^2^ against ALL reflections. The weighted *R*-factor *wR* and goodness of fit *S* are based on *F*^2^, conventional *R*-factors *R* are based on *F*, with *F* set to zero for negative *F*^2^. The threshold expression of *F*^2^ > σ(*F*^2^) is used only for calculating *R*-factors(gt) *etc*. and is not relevant to the choice of reflections for refinement. *R*-factors based on *F*^2^ are statistically about twice as large as those based on *F*, and *R*- factors based on ALL data will be even larger.

### Fractional atomic coordinates and isotropic or equivalent isotropic displacement parameters (Å^2^)

**Table:**

	*x*	*y*	*z*	*U*_iso_*/*U*_eq_	
U1	0.45262 (4)	0.79563 (9)	0.83956 (3)	0.0174 (2)	
U2	0.54899 (4)	1.08385 (9)	0.65373 (3)	0.0172 (2)	
Cl1	0.3096 (3)	1.0768 (5)	0.91681 (19)	0.0219 (8)	
Cl2	0.6957 (3)	0.8008 (6)	0.5776 (2)	0.0228 (8)	
O1	0.5897 (9)	0.8918 (15)	0.8900 (5)	0.023 (2)	
O2	0.3157 (9)	0.6944 (16)	0.7847 (5)	0.020 (2)	
O3	0.5411 (9)	0.4358 (15)	0.8468 (5)	0.020 (2)	
O4	0.3839 (10)	0.5906 (16)	0.9501 (6)	0.029 (2)	
O5	0.6817 (9)	1.1917 (16)	0.7088 (5)	0.021 (2)	
O6	0.4135 (9)	0.9824 (15)	0.6013 (5)	0.020 (2)	
O7	0.6135 (10)	1.2896 (16)	0.5424 (6)	0.028 (2)	
O8	0.4552 (9)	1.4395 (16)	0.6490 (5)	0.021 (2)	
O9	0.5643 (8)	0.7649 (17)	0.7297 (5)	0.019 (2)	
O10	0.4367 (9)	1.1133 (14)	0.7628 (5)	0.018 (2)	

### Atomic displacement parameters (Å^2^)

**Table:**

	*U*^11^	*U*^22^	*U*^33^	*U*^12^	*U*^13^	*U*^23^
U1	0.0166 (4)	0.0182 (4)	0.0173 (3)	0.00014 (19)	0.0014 (2)	0.00002 (17)
U2	0.0173 (4)	0.0174 (4)	0.0169 (3)	−0.00055 (19)	0.0016 (2)	−0.00013 (17)
Cl1	0.0245 (19)	0.0218 (18)	0.0200 (17)	0.0052 (14)	0.0052 (14)	−0.0007 (13)
Cl2	0.0264 (19)	0.0220 (18)	0.0205 (16)	0.0029 (14)	0.0048 (14)	−0.0024 (13)
O1	0.027 (6)	0.019 (5)	0.023 (5)	0.004 (4)	0.003 (4)	−0.001 (4)
O2	0.025 (5)	0.021 (6)	0.014 (4)	−0.007 (4)	0.000 (4)	−0.003 (4)
O3	0.021 (5)	0.016 (5)	0.022 (5)	0.009 (4)	−0.002 (4)	0.002 (4)
O4	0.028 (6)	0.026 (6)	0.034 (6)	0.006 (5)	0.012 (5)	0.003 (4)
O5	0.015 (5)	0.026 (6)	0.021 (5)	−0.011 (4)	0.000 (4)	0.000 (4)
O6	0.020 (5)	0.017 (5)	0.023 (5)	−0.002 (4)	−0.006 (4)	0.002 (4)
O7	0.031 (6)	0.022 (6)	0.032 (6)	−0.011 (5)	0.004 (5)	0.004 (4)
O8	0.019 (5)	0.022 (5)	0.021 (5)	0.003 (4)	−0.004 (4)	0.004 (4)
O9	0.013 (5)	0.023 (5)	0.019 (5)	−0.008 (4)	−0.007 (4)	0.003 (4)
O10	0.023 (5)	0.011 (5)	0.018 (5)	−0.004 (4)	−0.002 (4)	−0.002 (4)

### Geometric parameters (Å, °)

**Table:**

U1—O1	1.746 (10)	U2—O6	1.759 (9)
U1—O2	1.789 (10)	U2—O5	1.772 (9)
U1—O10	2.367 (9)	U2—O9	2.366 (10)
U1—O9	2.382 (9)	U2—O10	2.373 (9)
U1—O3	2.397 (9)	U2—O8	2.396 (10)
U1—O4	2.490 (10)	U2—O7	2.488 (10)
U1—Cl1	2.751 (3)	U2—Cl2	2.772 (3)
U1—U2	3.9492 (10)		
			
O1—U1—O2	177.7 (4)	O6—U2—O9	90.9 (4)
O1—U1—O10	91.5 (4)	O5—U2—O9	89.3 (4)
O2—U1—O10	87.9 (4)	O6—U2—O10	89.8 (4)
O1—U1—O9	88.8 (4)	O5—U2—O10	88.2 (4)
O2—U1—O9	88.9 (4)	O9—U2—O10	67.4 (3)
O10—U1—O9	67.2 (3)	O6—U2—O8	88.9 (4)
O1—U1—O3	88.5 (4)	O5—U2—O8	89.6 (4)
O2—U1—O3	90.6 (4)	O9—U2—O8	140.9 (3)
O10—U1—O3	142.3 (3)	O10—U2—O8	73.5 (3)
O9—U1—O3	75.2 (3)	O6—U2—O7	92.3 (4)
O1—U1—O4	93.8 (4)	O5—U2—O7	88.7 (4)
O2—U1—O4	87.8 (4)	O9—U2—O7	148.9 (3)
O10—U1—O4	148.5 (3)	O10—U2—O7	143.5 (3)
O9—U1—O4	143.9 (3)	O8—U2—O7	70.1 (3)
O3—U1—O4	68.9 (3)	O6—U2—Cl2	90.1 (3)
O1—U1—Cl1	91.0 (3)	O5—U2—Cl2	92.1 (3)
O2—U1—Cl1	91.1 (3)	O9—U2—Cl2	75.4 (2)
O10—U1—Cl1	76.0 (2)	O10—U2—Cl2	142.7 (2)
O9—U1—Cl1	143.1 (3)	O8—U2—Cl2	143.8 (2)
O3—U1—Cl1	141.7 (2)	O7—U2—Cl2	73.7 (3)
O4—U1—Cl1	72.9 (2)	O6—U2—U1	90.7 (3)
O1—U1—U2	90.0 (3)	O5—U2—U1	88.3 (3)
O2—U1—U2	88.3 (3)	O9—U2—U1	33.8 (2)
O10—U1—U2	33.6 (2)	O10—U2—U1	33.5 (2)
O9—U1—U2	33.6 (2)	O8—U2—U1	107.0 (2)
O3—U1—U2	108.7 (2)	O7—U2—U1	175.8 (2)
O4—U1—U2	175.4 (3)	Cl2—U2—U1	109.21 (8)
Cl1—U1—U2	109.56 (7)	U2—O9—U1	112.6 (4)
O6—U2—O5	177.8 (4)	U1—O10—U2	112.9 (4)
